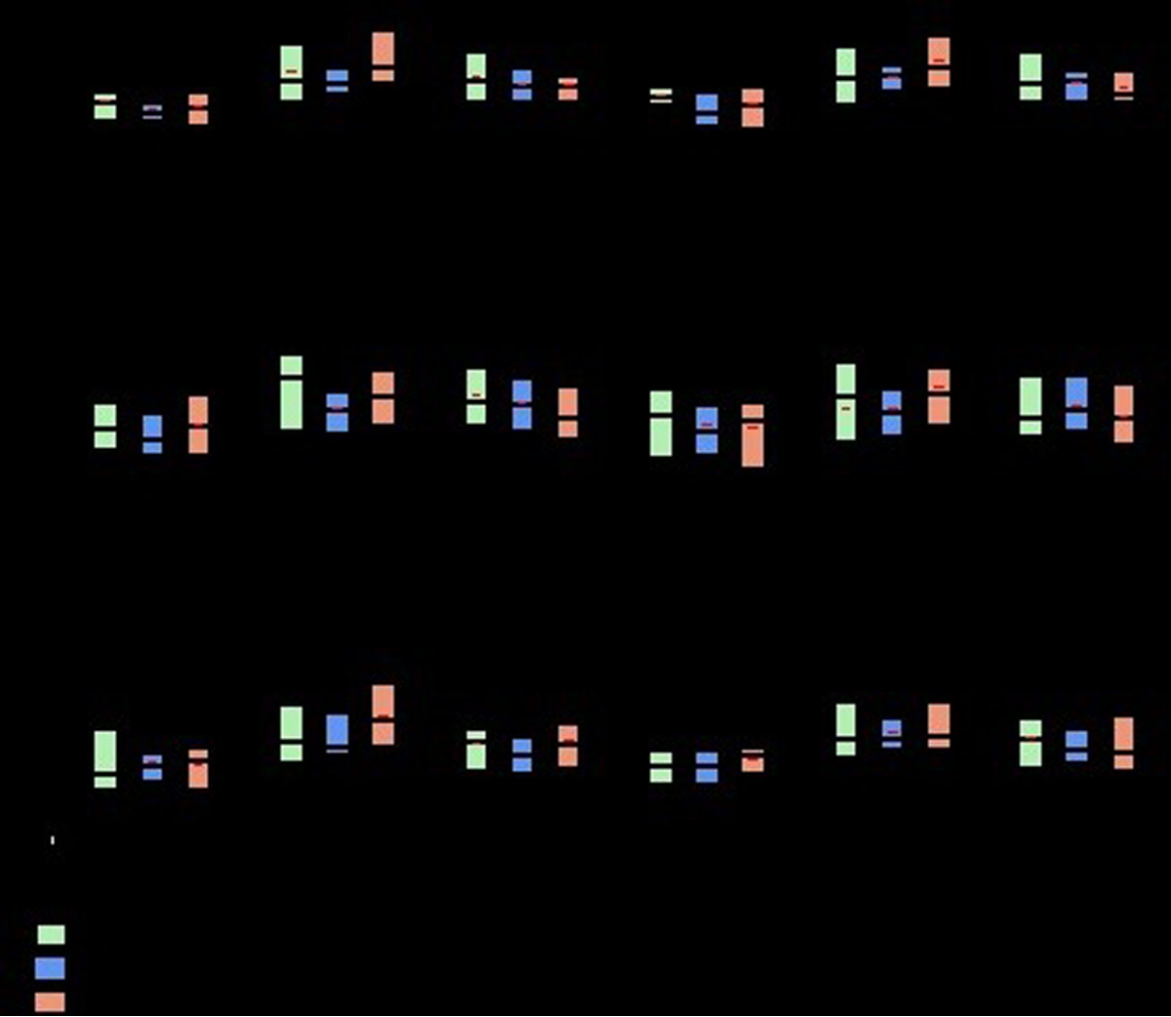# CMR left atrial characterization in Cushing's syndrome: a feature tracking study

**DOI:** 10.1186/1532-429X-17-S1-P42

**Published:** 2015-02-03

**Authors:** Roux Charles, Morgane Evin, Peter Kamenicky, Elie Mousseaux, Zainab RAISSUNI, Jerome Lamy, Phillipe CLUZEL, Nadjia Kachenoura, Alban Redheuil

**Affiliations:** 1 Pierre et Marie Curie Paris VI France, Paris, France; 2 Hôpitaux universitaires de Paris Sud, Paris, France; 3 Université Descartes, Paris V, Paris, France; 4 Faculté de médecine de Tanger, Tanger, Morocco

## Background

The aim of this study was to automatically assess global and regional left atrium (LA) strain in Cushing's syndrome (CD). Modifications of LA and LV function in patients have been highlighted followed by the restoration of myocardial function after radical treatment and cortisol normalization. Our hypothesis was that a feature-tracking method could improve the understanding of the mechanisms of LA alteration concerning its reservoir, conduit, and atrial contraction phases.

## Methods

Eighteen patients (37±13 yrs) diagnosed with Cushing's syndrome underwent comprehensive CMR including 3 long axis cine SSFP views before and after neurosurgical treatment and were compared to 18 controls (39±16 yrs) matched on gender and body size. Regional and global LA functional parameters were measured using a validated custom CMR feature tracking algorithm. Longitudinal strain and strain rate, radial motion fraction and radial relative velocity were quantified. The LA volumes and LA ejection fraction were measured using the QMass® V6.0 (Medis, Netherlands) dedicated software.

## Results

No differences were found in left atrial tele-diastolic volume (LADV) between patients and controls. LA ejection fractions were significantly reduced in patients (p<0.001) and increased significantly after treatment (p=0.001). Reservoir left atrial function was impaired: in CD, longitudinal strain rate (systolic peak: 1.4±0.8%/s vs. 1.1±0.4, p=0.02) and radial relative velocity (systolic peak: 1.5±0.8 vs. 1.1±0.5, p = 0.001) were also significantly decreased. The conduit function was also impaired as depicted by both longitudinal strain rate (-1.4±0.8 vs -1.2±0.5, p= 0.08) and radial relative velocity (-1.5±0.7 vs. -1.1±0.6, p =0.001). LA contraction was decreased in CD as compared to patients in radial relative velocity (-1.7±0.9 vs. -1.3±0.7; p<0.04).

After treatment, reservoir radial relative velocity tended to correct although not reaching significance. LA contraction function improved significantly in CD as longitudinal strain was increased from 10.4±4.7 to 12.9±6.9, p=0.002. Interestingly although LA reservoir and contraction functions increased after treatment, there was no improvement of conduit function. Segmental analysis of LA contraction highlighted non homogenic LA functional recovery, with increase of anterior-septal longitudinal strain (p=0.04) and septal radial motion fraction (p<0.02) during reservoir and LA contraction phases.

## Conclusions

A left atrial feature tracking method enables further understanding of LA alterations in Cushing's syndrome by showing both global and regional dysfunction. Reservoir, conduit and contraction phases of LA function were impaired in CD and both reservoir and contraction improved after treatment while conduit did not correct, highlighting the issue of left ventricular filling pressures in CD.

## Funding

N/A.

**Figure 1:**